# Nutrition and dementia care: developing an evidence-based model for nutritional care in nursing homes

**DOI:** 10.1186/s12877-017-0443-2

**Published:** 2017-02-14

**Authors:** Jane L. Murphy, Joanne Holmes, Cindy Brooks

**Affiliations:** Faculty of Health and Social Sciences, Royal London House, Bournemouth University, Christchurch Road, Bournemouth, BH1 3LT UK

**Keywords:** Nutrition, Dementia, Residential care, Eating, Drinking, Meal environment, Qualitative

## Abstract

**Background:**

There is a growing volume of research to offer improvements in nutritional care for people with dementia living in nursing homes. Whilst a number of interventions have been identified to support food and drink intake, there has been no systematic research to understand the factors for improving nutritional care from the perspectives of all those delivering care in nursing homes. The aim of this study was to develop a research informed model for understanding the complex nutritional problems associated with eating and drinking for people with dementia.

**Methods:**

We conducted nine focus groups and five semi-structured interviews with those involved or who have a level of responsibility for providing food and drink and nutritional care in nursing homes (nurses, care workers, catering assistants, dietitians, speech and language therapists) and family carers. The resulting conceptual model was developed by eliciting care-related processes, thus supporting credibility from the perspective of the end-users.

**Results:**

The seven identified domain areas were person-centred nutritional care (the overarching theme); availability of food and drink; tools, resources and environment; relationship to others when eating and drinking; participation in activities; consistency of care and provision of information.

**Conclusions:**

This collaboratively developed, person-centred model can support the design of new education and training tools and be readily translated into existing programmes. Further research is needed to evaluate whether these evidence-informed approaches have been implemented successfully and adopted into practice and policy contexts and can demonstrate effectiveness for people living with dementia.

## Background

The growing prevalence of dementia worldwide in our aging society has been widely reported with estimates of about 65 million people having dementia by 2030, rising further to 113 million by 2015 [1, 2]. Complex nutritional problems arise in dementia over the course of the disease with the progressive decline in cognitive and behavioural functions, finally losing the ability to independently function physically [3]. Thus due to mental and cognitive impairments, physical disabilities and psychological factors (depression and agitation), people living with dementia have difficulties associated with eating and drinking. These include partial or complete help and support required to eat as skills may be lost (access food to mouth), decreased sense of thirst and the ability to chew. People with dementia may experience increased appetite, rapid eating and repeatedly asking for food or have compulsive eating needs associated with some types of dementia. Dysphagia may develop and is reported in 13–57% of people with dementia [4]. Other mealtime behavioural challenges that affect intake include wandering, pacing, refusal behaviour, apathy or indifference. In addition to the dementia-related problems, people with dementia may also be affected by age-related comorbidities and taken together these can exacerbate reductions in intake and undernutrition [5–7].

In the UK the majority of people with dementia live in the community, and this care is provided by formal or informal care givers (family carer or relative) [6]. Nutrition-related complications in dementia can contribute to stress and caregiver burden. In addition this burden can develop into a cycle that can increase the risk of poor eating behaviour [8] and weight loss [9, 10]. Nutritional interventions that enable improvements in food and drink intake offer an opportunity to interrupt the potential risk of weight loss, undernutrition and dehydration, the consequent decline in cognition as well as alleviating the associated care burden.

A number of recently published articles have reviewed the current research on interventions aiming to improve food and drink in people with dementia including those living in long term residential care [11–14]. Interventions that modify food and drink include—oral nutritional supplements, assistance with eating and drinking, managing swallowing problems as well as those that offer environment-related interventions during mealtimes including eating location and arrangement, ambient sounds and music, aroma, temperature and lighting and food presentation. However there is no definitive evidence on the effectiveness and sustainability of such interventions to improve the nutritional health and wellbeing of people living with dementia in long term care settings or the potential to reduce cognitive decline in dementia [11–13]. Liu et al. [15] highlight concerns that many training programmes and mealtime assistance interventions in original studies are implemented by trained research assistants or researchers instead of front-line nursing care staff. This could contribute to the lack of effectiveness observed in many studies. Although there is a growing body of research for strategies to improve person-centred food and drink intake in people living with dementia, many existing approaches have not been tested in real-world settings with all those responsible for the delivery of care [16]. Therefore the aim of this study was to provide a research informed model of day-to-day application gathered from multiple perspectives to inform and upskill those responsible for delivering food and nutrition for people to continue living with dementia in care homes. In this way it will be possible to comprehensively shape which aspects are primary targets for further interventions.

## Methods

This qualitative study obtained rich contextual data using focus groups and semi-structured interviews. Participants were purposefully sampled from care homes and healthcare services in the community and carer groups from a local database of care providers specialising in dementia care. In total 27 care organisations were identified across rural and urban locations and care staff invited to participate in the study. Participants represented all those involved in the care of people living with dementia including care staff (health care assistants, nurses, care home and hospitality manager, catering staff) family carers, dietitians and speech and language therapists.

### Data collection

The interview guide was developed by reviewing other qualitative research exploring eating and drinking in people living with dementia in community settings to inform questions that would elicit the most informative responses. This was then discussed with the research team and key stakeholders on the project steering group (comprising a commissioner, lay user, workforce development representative, councillor representing social care) to determine what questions would most thoroughly explore the participants’ experiences.

Topics and questions included the following:

- Tell me about your experiences of providing meals and drinks to residents/family member
- What do you enjoy about providing meals and drinks to residents/ family member?
- What do you find difficult about providing meals and drinks to residents/ family member?
- What would help you in providing meals and drinks to residents/family member?
- How do you think these areas could be improved?
- What issues do residents with dementia confront when eating and drinking?
- What happens when residents don’t like meals or drinks? How can this be improved?
- How to make food and drink more familiar to residents/ family member? Are there associations with their past? How is it presented? How it is delivered: plate, bowl and cutlery?
- How do residents’ cultural and religious sensitivity affect how they see different foods and drinks?

Focus groups were held in a neutral venue led by the research assistant (CB) for this project with background experience of qualitative methods and prior research experience on a project involving people and staff in healthcare and community settings. Two researchers (CB and either JH or JM) were present in each group or semi-structured interview with the research assistant (CB) leading the discussion each time. At each focus group and interview she was supported by one other researcher to moderate and for quality control purposes.

All focus groups and interviews were recorded and transcribed verbatim to ensure accuracy but anecdotes or jokes were not taken into account. Only comments relevant to the research question were transcribed.

Ethical approval for the study was obtained from Bournemouth University Research Ethics Committee. Informed written and verbal consent was provided throughout the study. Participants were not obliged to participate and had the opportunity to ask questions before the sessions took place.

### Data analysis

The data collection and analysis was an iterative process, using Braun and Clarke’s [17] six-stage method of thematic analysis, and ultimately leading to data saturation. In the first stage, the analysis started with familiarization with data, through reading and rereading the transcripts. This was followed by the generation of coding nodes and features of the data were coded across all the transcriptions. In stage three, initial codes were grouped together which allowed the explanations to be identified. Themes were cross-checked by the research team for agreement to minimise bias. Overarching themes that grouped the initial codes were developed to create a framework for writing up the analysis in stage five. Stage six involved writing up the analysis and selecting extracts to illustrate themes.

Names of participants were replaced with codes to assure anonymity. It was noted if a participant agreed to the comment of another participant. To improve the rigour of the analyses and the trust-worthiness, we introduced a number of strategies including the coding and ordering process of excerpts in the transcripts. Triangulation of researchers was performed: all transcripts were first analysed by two researchers independently (CB and JH).

The coding and the interpretation of the codes were then discussed by these two researchers to deepen their analyses and to reach consensus about what were main themes. In addition, the other author (JM) read and analysed at least one transcript. All authors commented on interim and final analyses of the data. The authors have different educational backgrounds (nutrition, food science, sociology).

## Results

### Participants

Nine focus groups and five semi-structured interviews were conducted with 50 participants. Family carers (*n =* 8) represented people living with dementia in long term care settings. Speech and language therapists (*n =* 9) and dietitians (*n =* 3) were all employed by the National Health Service. There were 30 care staff recruited from 20 care home organisations. Of these 26 participants (87%) worked in care home facilities that had more than 20 residents. The majority of the participants were female. Details of the participants are shown in Table 1.

**Table 1:** Details of the participants

	Care staff	Family carers	Dietitians	Speech and Language Therapists
Number of participants	30	8	3	9
Gender % female	93	17	100	100

### Key themes

Overall seven themes emerged from the data (Table 2) that were inter-related but were not mutually exclusive.

**Table 2:** Themes and sub-themes derived from focus groups and interviews

Themes	*Sub-themes*
Person-centred nutritional care	*Stage of dementia* *Psychosocial factors* *Life histories* *Health conditions* *Generational factors* *Cultural factors*
Availability of food and drinks	*Accommodating changing tastes and preferences* *Presentation of meals* *Modification of food e.g. food fortification and using food* *purees* *Supplements* *Prioritisation of food and drink* *Delivery, prompting and offering a drink*
Tools, resources and environment	*Nutrition screening* *Modified equipment* *Ready plated meal choice options* *Contrasting colours* *Constant prompting/encouragement/giving time to person* *Environmental factors: Setting the table*
Relationship to others when eating and drinking	*Relationship to family members* *Relationship to care staff* *Relationship to other residents*
Participation in Activities	*Create aromas to stimulate appetite and evoke memories* *Enhance appetite and sense of purpose and identity* *‘Themed days’*
Consistency of care	*Prioritisation of nutrition and hydration* *Improved communication between all those involved in care*
Provision of Information	*Access to trusted information and resources* *Education and training* *Current guidelines*

The overarching theme of *‘person-centred nutritional care’* identified the need to prioritise the nutrition and hydration needs and preferences of people living with dementia. The second theme related to the importance that food and drink should be readily available, entitled ‘*availability of food and drinks’.* The third theme, ‘*tools, resources and environment’* focused on the range of tools, resources and influential environmental factors needed to support the delivery of food and hydration. The fourth theme, ‘*relationship to others when eating and drinking’* was concerned with how the presence of others, including care staff, family members and other residents and the setting (own room, dining room and other communal area) affected the person’s mealtime experience. The fifth theme, ‘*participation in activities’* captured the ways in which activities could be used to engage residents and stimulate the appetite. The sixth theme, ‘*consistency of care’* recognised the need for prioritisation and consistency in the provision of nutritional care for people living with dementia in care homes but also across health and social care environments (at home, across care sectors – care homes, in hospital, day centres). This led on to the final theme, ‘*provision of information’,* that embraced the need for better information, education, training and support to guide nutritional care for both formal and informal carers.

Each theme is described in turn below and illustrated through verbatim representative extracts for either focus group or interview. The extracts are labelled according to the type of participant: care staff (CS), dietitian (DT), family carer (FC), speech and language therapist (SLT), director of nursing (DN), hospitality manager (HM).

### Person-centred nutritional care

Participants reported that recognition of the stage of dementia was essential to provide appropriate support to meet nutritional needs and preferences. As dementia progresses recognition of eating utensils, food and dining environment starts to diminish and there is a reduced understanding of mealtimes. Participants reported that as dementia develops it became difficult to persuade people to eat:

‘There’s the early dementia where those markers [meeting somebody's full nutrition and hydration requirements] are achievable and there’s a point at which those markers just aren't. And I think that’s really hard for the care staff and the families, but I’ve got to make him eat, I’ve got to, he’s got to eat and there’s this kind of force-feeding. That’s where I think there’s a gap in the literature we give people.’ (focus group 9, SLT 2)

Participants reported that due to changes in the provision of care, inadequate staffing levels were increasingly problematic. In the past, generally people living with dementia may have come into the care home at an earlier stage if not possible to be cared for in the community at home. They are coming into a care home at a later stage of their dementia journey when their nutrition and hydration preferences and needs may have significantly changed requiring more support, patience and time from care staff:

‘across the board dementia care is way under-staffed because we’re seeing such higher dependency of people now coming in. People with dementia who traditionally maybe would have come into care homes much earlier are now being cared for at home in the community … it’s really difficult because you might have one member of staff that’s trying to support four people to dine well and it’s quite impossible…’ (focus group 7, CS 2)

Participants acknowledged the variability in psychosocial factors including a person’s mood, whether their preferred carer had come that day and the time of day could impact upon a person’s responsiveness to eating. If those living with dementia were anxious or worried about something then this would affect their hunger patterns and willingness to eat. The motives that underpin these responses required more understanding to settle residents to be encouraged to eat and drink. This might include reassurance or changing the environment in which they were eating:

‘…and it could be psychological couldn't it, reasoning, people give for dementia if they’re particularly anxious that day, if something’s frightened them or worried them, they may not know what that is … some of us will when we’re worried, eat and others don’t want to eat, and that’s not uncommon, we see that a lot.’ (focus group 3, CS 3)

It was recognised that the person’s responsiveness was related to whether he/she was clean and in a comfortable position to support their dignity:

‘Y’know the resident may no longer be able to ensure they maintain their dignity, but y’know the staff do that. They make sure their hands are clean before they take their meal and that their hands and their face and their clothing is clean … A lot of these residents have very upstanding professions, livelihoods, y’know characters, experiences and it doesn’t mean because they can no longer do it you just ignore it now y’know. It’s very much instilled the dignity side of things.’ (interview 2, DN 1)

Knowing a person’s life history including their relations to family and friends, their former occupation, whether they have lived abroad, which languages they spoke, whether they have recently lived alone or with other(s) were identified as integral to understanding and supporting a person’s nutritional needs and preferences:

‘we had a lady that came into this home and she’d been diagnosed with dementia… her main meal would be put in front of her and the first thing she would do is actually tip one of the drinks she had beside her, whether it be squash or wine or whatever on to her meal … but we then chatted to the family and we actually discovered this lady had been a horticulturalist in her working time, and the plates that we used at that time had a rim of flowers … she wasn’t seeing the meal … she was seeing the flowers and she was watering the flowers. And the minute we changed our crockery, we tried it initially with her on to a plain white plate, it stopped.’ (focus group 5, CS 3)

Importantly, food was recognised as occupying one of the last functions that people living with dementia may influence. The following example reveals food can occupy a way of exercising agency and expressing decision making:

‘and it’s like food is the last thing many people have any sort of control over, it’s almost like the anorexic, bulimia sort of cycle of I can’t control where I am or what I do or what people are making me do but I can mmm decide what not to eat or not eat lots.’ (focus group 3, CS 2)

Participants acknowledged how a range of health conditions, such as infection, sensory loss, mobility issues, dental problems or ill-fitting dentures and the side effects of medication should be carefully monitored by care staff. Close observation and monitoring of these factors is especially important as dementia progresses and people may not be able to communicate pain, discomfort or anxiety when eating and drinking:

‘the important things with dementia is that they can’t tell you that they’re not eating. You need to look for their physical things first. They might just be unwell or they might have a sore throat… to be careful of their dental hygiene as well. Ill-fitting dentures, things like that really have to sort of eliminate those things first.’ (focus group 4, CS 1)

Generational factors were recognised as important in influencing a person’s nutritional and hydration preferences. Food and drink preferences earlier in their life may become more prominent. For instance participants indicated that the age range within one home ranged between 60 and 104 years. They had introduced a range of food to accommodate changing generational tastes, for example moussakas, cannelloni, sweet and sour chicken.

Religious and cultural background can also influence nutritional and hydration preferences. Although as a result of dementia, a person may express an appetite for food types which are prohibited within their religion. This might create discordance between the care staff wishing to respect the resident and their family’s religious dietary practices but also responding to the resident’s appetite and desire for certain food types:

‘we’ve always respected and taken on board any faith/cultural background…I think we’re still somewhat sheltered at the minute, maybe the next generation we’ll start to see that mixture…a lot of our older gentlemen have been abroad in their younger years and you can see where perhaps y’know contrary to what their wife might think they might actually love a spicy food.’ (interview 2, DN 1)

Participants reported how there was a need for better nutritional training and education (see later) but particularly for chefs around food fortification to improve energy intake and the presentation of modified meals such as purees including the use of food moulds. The appropriateness of which foods to puree to meet person needs eg pureeing of meal components to maximise retention of colour and aroma were also identified:

‘For our chef…the minute he made the first batch he was so happy that he could actually provide somebody who needed the consistency of diet, something that looked…dignified and we’ve never looked back…Y’know you’re not talking of the average older population, you’re talking about the most vulnerable…highest dependent need and you’ve got to take that on board.’ (interview 2, DN 1)

The following six interconnected themes are discussed and in relation to person-centred nutritional care where relevant.

### Availability of food and drinks

Participants reported that food and drink should be readily available, easily accessible and offered to residents throughout the day and night. Whilst many care homes provided menus, participants stressed that menus should be used flexibly in accordance to whether they are fulfilling nutrition and hydration needs and preferences and ensuring cultural diversity. Where these needs were not supported by the menu, participants emphasised how other food and drink choices should always be available. In particular, when people with dementia elicited signs of hunger, food and drink should be offered with encouragement, patience and calm.

‘If they say we need some eggs and bacon because… the resident wants this at this time of the morning…the staff are good because we explain… but they’ve got an appreciation of what that person likes in the day or what a typical behaviours are of the day and they can take that on board on nights…’ (interview 2, DN 1)

Participants reported changes in people’s tastes as dementia advances, including a preference for stronger flavours, such as sweet, salty, spicy and sour foods. These can be accommodated by adding spices and seasoning to increase the strength of flavours:

‘..and if you educate them, if you actually talk them through then obviously advise people to actually have condiments racks in the room that they will add the salt and pepper and paprika and herbs actually on the meal as it comes in but it’s not something that the homes feel particularly confident, I don’t think in doing off their own back I don’t think they know that bit.’ (focus group 9, SLT 3)

The time of day was also identified as affecting a person’s appetite, with participants identifying the need to engage people when most alert, interested and awake:

‘My experience is … breakfast and the lunches that’s mainly when they’re interested, then after lunchtime it gets harder and harder for people to, to encourage people to eat and most of our residents would find it difficult to choose.’ (focus group 2, CS 2)

In comparison to formalised mealtimes which may place more expectation upon residents to eat, participants reported how the ready availability of a variety of meal options including frequent offering of smaller or ‘mini-meals’, grazing food and snacks in different settings gave people flexibility to eat when hungry or motivated to:

‘Finger food, definitely. As the dementia progresses we tend to see that massively increases because they want to retain as much independence as possible.’ (focus group 2, CS 2)

Participants stressed the importance of paying careful attention to stimulating the senses including colourful food, attractive presentation and use of aromas:

‘they know it’s fish and chips because of the smell of fish and chips, I mean the aroma…and it’s also like the aroma makes you hungry so if a client doesn’t want to eat and you say well I’ve got a nice bowl of carrot soup…you can smell the beautiful spices or the smell of carrot.’ (focus group 5, CS 3)

Participants emphasised the importance for accounting for changes in a person’s appetite and ability to swallow.

‘..and when we started looking…it was often misuse with dentures and other things rather than actually a problem of swallowing… we were in a situation where even when we got the dentures and everything fixed that person had not had solid food for so long… we did have to get a SALT (speech and language therapist) referral done because they couldn’t swallow, everything had changed here and they literally did struggle. (focus group 1, CS 1)

In addition professional support and advice from dietitians and diabetes nurse specialists about ways to modify existing foods to reduce the sugar content for people living with dementia and diabetes was reported:

‘we don’t have any diabetic products in our kitchens at all. We advocate that if someone is, has a normal type 2 diabetic then they have little bits, maybe, we might limit the amount of sugar, but we don’t start dishing out diabetic jam, diabetic pudding, low fat cake… it’s all of the same, just maybe a little less of it, and again that’s down to encouraging them.’ (interview 3, HM 1)

Oral nutritional supplements were reported by participants as a way of boosting nutritional intake but there had been a notable reduction in these being prescribed to residents. It was noted smoothies and milkshakes were used to replace these and enhance energy intake:

‘we’ve made a significant reduction in the amount of supplements that people have, partly that’s been driven by the GPs not being so willing to prescribe it because it’s a cost,….. they’ve been having with more smoothies and shakes and milkshakes which again because they’re sweet the residents tend to really enjoy.’ (interview 3, HM 1)

Due to the loss of thirst sensation, participants identified how hot and cold drinks should be readily available to residents in a variety of settings day and night. Drinks should be offered irrespective of whether residents had recently had a drink.

‘we’ve got one lady whose out and about all day. She walks around and however she prefers tea and coffee, she’s not much of a cold drink girl, so we provide more tea and coffee to her y’know. It’s about what they want… and then carers just go in and encourage fluid intake throughout the day.’ (focus group 4, CS 1)

Participants reported a variety of options for increasing residents’ fluid intake including ice lollies, jellies, sundaes, cool drink and water machines, smoothies, mini cartons, juice fruit, melons, oranges, cucumbers, spring onion, salad and fruit bowls. As the following quote illustrates these options were thought to be especially important in warmer temperatures:

‘..but some days you have orange juice, blackcurrant juice, different juices and it’s…somebody just gets fed up with every day the same food or the same drinks.’ (interview 1, CS 1)

The importance of encouragement, prompting and patience when offering a drink was identified by this family carer:

‘If you ask him if he wants a cup of tea he will have one but unless somebody’s going to keep asking him every half an hour do you want a cup of tea, I doubt if he’d get one.’ (interview 4, FC 1)

### Tools, resources and environment

The use of the Malnutrition Universal Screening Tool ('MUST') [18] was identified as a way of identifying whether residents were at risk of malnutrition:

‘…we kind of document that into a care plan and ‘cos we’ve got nutritional care plans and we use the MUST tool and I’ve got a carer who weighs people every month, but if they lose weight then we put them on a special chart and then we weigh them more often and so.’ (focus group 3, CS 4)

Using the colour codes of red, amber and green to alert kitchen staff and make all other care staff aware of those at risk of undernutrition was highlighted:

‘it’s a colour-coded system… the kitchen staff know that anybody with a red coloured code is, they need to keep a real close eye on those. Amber is yeh OK, and green are low. We just use that as a visual tool.’ (focus group 7, CS 4)

A range of tools such as plate guards, modified cutlery and beakers can be particularly helpful in supporting residents nutritional and hydration needs. As dementia progresses, sometimes having less tools help the person to focus on eating and drinking more easily:

‘Finger food, definitely. As the dementia progresses we tend to see that massively increases because they want to retain as much independence as possible, they tend to prefer food they can access easily so soups they’ll suddenly stop eating, but yeh if you pop like a really nice display of finger food in front they’ll all quite happily sit there and eat those.’ (focus group 7, CS 2)

Visual aids such as picture cards and flashing screens could be useful to depict meals, these were recognised as unsuitable especially as dementia advanced. Due to memory loss, cognitive changes and sensory deterioration associated with dementia, identification of food through photographs became more difficult with consequent trouble remembering what had been shown.

A recurrent finding was the disadvantage of using menus as memory loss associated with dementia leads to the inability to recall selection of meals. Rather than visual aids, a number of participants emphasised how presenting residents with ready plated meals on a tray at meal times and patiently offering alternatives if appropriate, was essential to addressing the memory, cognitive and sensory issues associated with dementia:

‘I don’t see that there is any better yet to me to show me than you showing me two plates of food and saying [name of interviewer] would you like this one or this one? This one’s the lamb and this one’s the chicken …Now if you, depending on the level of dementia and type of dementia you might take quite a long time to choose, it might take you several minutes, you might still when [name of resident] dinner turns up decide you want that one… I think choice has got to be on the plate, in front of you, see it and smell it, have a feel if you want, have a poke of it, but not pictures. Definitely not.’ (interview 3, HM 1)

Contrasting colours of crockery, enabled identification of food items but they should represent regular household utensils to increase the familiarity to the resident:

‘we use the red sensation that actually looked like real crockery and you have to go up to it and pick it up to notice that it’s not, it’s got like a fleck through it black and silvery fleck with a sheen over the top so it looks like porcelain.’ (focus group 3, CS 2)

With respect to utensils for serving drinks, participants acknowledged that whilst utensils should be adapted and able to support the needs of the person they should look as much like everyday household utensils to appear familiar and support a person’s dignity:

‘Some people couldn’t pick up that mug so it’s about kind of adapting so we’ve bought plastic ones with two handles, we’ve bought camping mugs because they’re lighter but still resemble a mug. It’s about normalising everyday utensils…’ (focus group 4, CS 2)

A range of environmental factors which could be used to support and engage the person during mealtimes were described. These included setting tables and the dining room in different ways for different meals. For example, providing tablecloths with flowers at lunchtime and using linen and napkins. Other environmental aspects included the creation of a relaxed atmosphere by using soothing background music, having sufficient space between tables, a comfortable temperature and the use of lighting and colours to evoke different atmospheres:

‘it’s far more about creating the right atmosphere y’know, all the basics, the lighting, the way the table is set up, background music, having the right implements, the food itself, I think all of these things are what’s creating a relaxed atmosphere.’ (interview 3, HM 1)

### Relationship to others when eating and drinking

Relationships needed to be assessed to suit the person with dementia. This aspect should be monitored and adapted according to the stage of dementia, mood, and whether the person with dementia prefers to eat and drink on their own and/or with others:

‘ It depends what stage of the dementia that they’re at because they need quite a lot of support and they need to be told to swallow so yes they need, if someone sits with them they’re more likely to have a meal than if someone, than if they’re just left to it.’ (focus group 6, SLT 5)

This theme is explored further to show the relationship with family members, care staff and other residents.

#### Relationship to family members

Responses were not consistent whether the presence of family member(s) (family carer/other relative/friend) sitting with the person encourages or inhibits the mealtime experience. A range of influential factors were identified including whether a person eats alone or with the family member, the stage of dementia and whether the family member is eating or drinking. Positive aspects of eating with a family member include familiarity, evoking past mealtime experiences and the benefits of encouragement to prompt eating and drinking. Negative aspects included placing unintentional pressure upon a person to eat, especially if a family member is not eating with the person.

‘some relatives are fantastic, amazing, but some have a limited understanding of dementia and how that can affect taste and appetite … and we try and explain to them things have changed, their needs and their tastes and y’know their abilities are changing‘ (focus group 7, CS 2)

#### Relationship to care staff

Staff eating with residents may help to afford trust especially if a person may have built up fears around eating and/or drinking. This practice can enable ‘copy-cat’ behaviours through the person imitating staff. On the other hand participants reported the potential for personality incompatibilities.

‘each carer can bring a different person out of themselves, I think personality-wise ‘cos it’s how they relate, how they engage with them definitely’ (focus group 5, CS 4)

#### Relationship to other residents

The relationship between residents was identified as contingent to a person’s stage of dementia, personality compatibility and whether they are used to eating on their own or with others:

‘the personalities on the table can have a major effect so you might have somebody who can’t abide noise or is quite noise sensitive, so if somebody’s particularly noisy if you sat them with that person it would probably result in them leaving the table so you’ve got to have a knowledge of your residents as you seat them.’ (focus group 7, CS 2)

### Participation in activities

A range of approaches through activities were described to engage residents and stimulate appetite. The use of aromas within the care home was acknowledged as an important route in building up anticipation of the meal and stimulating the senses. These included aromas of cooked breakfast and baking, buying artificial aromas such as coffee, cherry almond bake and fresh bread and the use of different aromas to signify different days, such as roast lamb on a Sunday morning. These aromas were identified to evoke past memories:

‘It’s amazing and just the smell of a cooked breakfast will start people talking about the cooked breakfast they remember they had after D-Day and you’ll get these amazing conversations y’know that come from just the aromas.’ (focus group 3, CS 3)

Interest in eating and drinking could be stimulated through involvement in activities that involved food (gardening, baking, going shopping, preparing meals, cake decorating). These activities could be tailored to the person’s stage of dementia, life history and past occupation.

‘it’s about stimulation, engagement, occupation so it can be things like y’know food preparation … it’s more about dexterity, it’s more about involvement, inclusion … engaging and doing something so they might be icing cakes … We look at our residents and we see what makes them tick and what they like to do and what we do [is] that with them. So some things can be one-to-one, some things can be group activities and it’s always up for changing.’ (interview 2, DN 1)

Themed days’ in the care home offered important opportunities to promote eating and drinking. These days included cultural awareness days involving a number of culturally related activities, ‘taster days’, celebratory events and occasions such as Christmas, birthdays in addition to regular events (cinema events with snacks, afternoon teas, ice cream days):

‘so we had a French-themed day one day and the chef came up with a beautiful variety of foods and one resident was eating and because the activity staff had put a beret and some onions on completely unprovoked, un y’know un-thought of, this resident started talking very fluently in French. … how important it is to capture all of the information about them because second-hand, third-hand guestimates from others with every best intention and will in the world will not extract the essence of that person.’ (interview 2, DN 1)

### Consistency of care

The importance of consistency in the provision and prioritisation of nutrition and hydration care for people living with dementia was widely recognised, supported by training, information and support. Better means of communication across the ‘circle of support’ (healthcare professionals -speech and language therapists, registered dietitians, GPs, nurses, care home staff, family carers, formal carers and people living with dementia) was integral to providing consistent care.

‘It needs to be spread over everything and not just from when [people] come to a care home. It needs to be in the day centres and in the homes, that’s the major problem.’ (focus group 3, CS 2)

Various strategies were proposed to support improvement of communication with participants emphasising how these should be initiated in the early stages of dementia, to ensure that their needs and preferences were being adequately met as their dementia advanced. These strategies included recording and sharing key information, (including life history, nutritional preferences, occupation), the introduction of multidisciplinary meetings, access to a Clinical Nurse Specialist or a Dementia Specialist Nurse to guide and navigate the person living with dementia to relevant services and resources and involvement of the family carer where appropriate.

This healthcare professional reported how an electronic patient record system between Dietitians and Speech and Language Therapists (requiring patient consent to share information), had improved communication between both parties.

’we have a new…electronic patient record system that both ourselves and speech and language therapy use …so as long as there has been some sort of consent from the patient to share their record then we can see what speech and language therapists have said and they can see what we have said.’ (focus group 8, DT 2)

Although sharing of information had become more difficult due to issues of confidentiality. As this example highlights:

‘but then that becomes an issue of confidentiality and consent and all the rest of it doesn’t it … do you remember that when we used to put communication plans above the bed, and oh no you can’t do that, you can’t … What about with your family member who took this journey?’ (focus group 9, SLT 2)

Temporary agency care workers and high turnover of staff made it difficult to follow-up or check information received leading to inconsistencies in care:

‘…and for the carers themselves they’re not sure how to approach that individual person to support them or think how they should be supported could be different to what happened on the previous day’ (focus group 8, DT 3)

The need for skilled dementia champions were identified to ensure that a person’s nutritional and hydration needs are being properly fulfilled and to prevent weight loss:

‘food…just being left in front of them and yet this person doesn’t know that you’ve got to lift the lid off to get to the food. Y’know they shouldn’t be drinking from like y’know a cup, like in their care plan they need to be drinking from a lidded beaker and y’know all these things they know that on some days when they’re having a good days, but they’re not having good days ‘cos they’re in hospital…on a bad day they need full assistance to dine, and that’s just not happening.’ (focus group 7, CS 2)

Participants stressed the importance of care staff communicating, encouraging and interacting with residents to build up residents anticipation of a meal. For example, the chef coming to have a conversation with residents to explain what meals are being served, using language that is appropriate for the resident. Patience and giving time to residents to understand and make choices were also identified as essential to helping residents feel comfortable and at ease:

‘Sometimes I find when our chef comes out and he’s obviously got all his chef uniform on it’s quite reminiscent of that, there’s a lot of sit up and oh this is just going to be just lovely and [he] comes back round and has a chat and I think it kind of puts them in a place oh, maybe we are in a restaurant find of thing…‘ (focus group 7, CS 6)

### Provision of information

Participants identified a need for trusted evidence based information, education and training about the provision of nutrition and hydration. Issues were associated with the volume of information available on the internet, inconsistent and conflicting advice and not knowing where to look. Participants emphasised a need for information to be in an accessible format to suit the learning style as well as resources to signpost and support relatives and family carers.

‘Yes I had to just learn by trial and error…It would have been nice to have had some knowledge. Y’know whether the tastes buds disappear… ‘ (interview 4, FC 1)

This evidence base should recognise changes in taste and swallowing which take place as a person’s dementia advances, the positioning of a person at mealtimes and associated issues of safeguarding:

‘… there’s a balance…and what are the best interests, is it y’know keeping them safe no matter what or is it actually acknowledging that they should actually have some enjoyment and pleasure from their food as well in the later stages of their life.’ (focus group 6, SLT 4)

As well as recognising the latest dietary guidelines regarding the importance of texture, taste and nutritional guidance:

‘And I think also y’know we’ve had experience recently of nursing homes, training staff supposedly in … dysphagia and dysphagia management *[…]* and by people working in the home who haven’t necessarily got the competency to do so and it’s kind of like tick in the box…whereas actually to what competency level and who’s been monitoring that’.. (focus group 6, SLT 3)

Importantly it was emphasised that any training and education provided needs to be monitored to determine if and how it is being implemented, to ensure the best quality care.

Model to inform strategies for the provision of good nutritional care in dementia

**Fig. 1:**
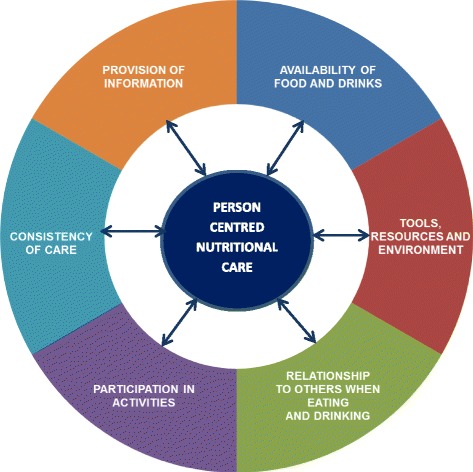
Model for the provision of good nutritional care in dementia

The experiences shared by the participants enabled the key themes to be constructed in the form of a model of good nutritional care (Fig. 1). At the centre of the model is the overarching theme to prioritise person-centred nutritional care. The determinants of person-centred nutritional care are influenced by the stage of dementia (and presence of other co-morbidities), psychosocial, cultural and generational factors.

## Discussion

The present study was undertaken to develop a model for understanding the provision of good nutritional care for people living with dementia in nursing homes. Our approach differs from that of previous studies that have explored eating and drinking from either the perspective of the formal or informal care giver [18, 19]. Specifically we developed this model empirically by engaging with stakeholders from multiple perspectives to identify care aspects and processes that could lead to improvements in the delivery of nutritional care in the real world.

Person-centred nutritional care was an overarching aspect of delivering nutritional care in dementia. The importance of ‘person-centred residential dementia care’ has been increasingly recognised over the last 20 years since the publication of Kitwood’s Seminal work, Dementia Reconsidered in 1997 [20, 21]. More specifically, there is recent literature showing the importance of mealtimes for families living with dementia highlighting strategies to promote meaningful mealtimes for family and how to stay socially engaged and continue mealtime routines and activities as dementia progresses [19]. Yet there would appear to be a lack of understanding about the role of eating and drinking and the meaning of the mealtime experiences of people living with dementia in the context of person-centred care. Thus the findings from the present study have illuminated a number of factors that directly contribute to person-centred care for nutrition in people with dementia that could be readily translated into existing programmes for providers of care.

The availability of food and drinks refers to the need for regular main meals and snacks. It recognises that energy needs may not be satisfied adequately through conventional food intake patterns and adaptations need to be made in response to differences in wandering behaviours, sleep patterns and physical activity. The present study offers a broader view of the traditional focus of the ‘meal experience’ in dementia with reference to three meals a day and to take a more holistic view of person-centred food and drink delivery over the whole day when people living with dementia are ready to eat [22]. Meals may need to be enriched with additional energy and protein, fortified snacks offered if weight loss has occurred, thus highlighting the need to identify those who have lost weight and at risk from under nutrition [23, 24]. The findings supported the need to modify food to encourage independent eating e.g. finger foods, use of mini meals, grazing menus particularly for people who elicit wandering patterns of behaviour [25, 26]. In some people food texture may require modification if there is difficulty chewing (though attention to dentition was emphasised as a common issue) and swallowing problems. [27] However the importance of maintaining dignity was highlighted and the use of innovative ways to improve the presentation of foods.

As sensory systems decline with aging, the findings support the need to present food and drink in an appealing and appetizing way in order to facilitate and encourage intake by working with a person’s preferences by boosting flavour, colour, taste and appearance [28, 29]. This aspect may also be determined by other diet-related issues e.g. food allergies, constipation or comorbidities e.g. type 2 diabetes.

As dementia progresses the decreased ability to remember to eat, to recognise food and eat independently and the need for constant negotiation and support during meals were reported. The findings support previous observations to encourage eating and drinking and that caregiver assistance should be tailored to suit a person’s needs, using verbal cues, positive reinforcement whilst helping to retain independent eating and ensuring dignity [19]. Studies have shown that increased time spent by care staff and nurses to support people during meals may positively affect eating behaviour, intake and nutritional status [25, 30].

The findings supported the use and application of nutrition screening [31], to identify those at risk of undernutrition and ensure a person-centred approach is taken to provide food and drink to people with dementia to improve intake. The use of appropriate resources and specialised equipment (e.g. adaptive crockery and utensils, no-spill cups) to support eating and drinking were recommended, with guidance offered by health care professionals as required. Previous studies support the use of high-contrast coloured crockery and tableware [32], though there is a lack of consensus of which type of colour to use to improve eating performance of food intake they point towards recognising a person’s preferences.

Environmental factors including eating location and arrangement, ambient sounds and music, smell, temperature, lighting and food presentation were reported to have an important role in reducing stress and anxiety at mealtimes and support previous observations [33]. These aspects have been the attention of recent systematic reviews [3, 15], that have described improvements in behavioural symptoms and dietary intake as a result of improved lighting, relaxing music and offering more homelike food service and environment.

The relationship to others when eating and drinking was another theme that emerged from the presence of others—other residents, staff and family members. This observation supports a previous study that demonstrates positive effects on body weight and eating behaviour in care units as a consequence of shared mealtimes between residents and care givers [34]. However the present study adds that it was important to recognise and respect a person’s preferences to eat alone and that preferences can change with the progression of dementia, mood and personalities.

The findings in the present study demonstrated the way in which ‘participation in activities’ around mealtimes can engage residents to evoke memories (e.g. through themed days, celebratory events). They can stimulate appetite through the physical nature of the activity e.g. gardening, preparing meals but also positively impact on quality of life. Thus these activities were key to engage residents with past memories and providing a sense of purpose and involvement whilst promoting a person’s independence and promote dignity. A previous study has shown that activity associated with living in ‘care farms’ can stimulate dietary intake through additional leisure and recreational activities [35]. There is also growing interest in the benefits of growing food and horticultural therapy as a means to improve physical and psychological wellbeing and social integration [36, 37]. Moreover behavioural interventions in residential long term care such as involving people with dementia in meal preparation have been shown to support social engagement and self-feeding [38]. However what has been revealed in the present study is the importance and benefits of providing and engaging residents in a wide range of food-related activities. More research is needed to understand how participation in activities can improve eating performance and food intake and the overall health and wellbeing of people living with dementia.

The prioritisation and consistency in the provision of nutritional care was reported by all those responsible for the delivery of food and nutritional care. The central importance of communication (both verbal and non verbal) and relationship building in the provision of compassionate care across all settings in general has been discussed in the literature [39]. However in the present study, the findings reveal the barriers evident to providing good nutritional care that could be attributed to poor communication between carers (both formal and informal), high staff turnover and poor record keeping.

The provision of evidence-based information on nutrition and hydration in an accessible format was clearly articulated by participants who discussed the need for education and training. Knowledge of nutrition in the course of the disease and on adequate intervention and communication is essential to provide appropriate nutritional care for people with dementia. There have been several studies that have tested teaching and training interventions for caregivers and nursing staff. They have shown positive effects of caregiver training and education with respect to knowledge and attitudes of caregivers and the nutritional situation of people with dementia [4]. Specifically certain interventions such as ‘Montessori methods’ and ‘spaced retrieval training’ have been shown to be effective in improving eating performance [40]. However the review by Liu et al. [15] showed that the duration of such approaches in studies were relatively short and implemented by trained research assistants/researchers and recommend that training should be undertaken by nursing caregivers (rather than research assistants) to improve eating performance.

We used several strategies to improve the quality of analysis and trustworthiness of results: triangulation of researchers, and discussions of interim and final analyses with authors who have different backgrounds. However the present study may be limited by collection of data across one regional area. The findings may not reflect the opinions and perceptions of those who work in other nursing homes or live in other parts of the country that would capture more diversity across different cultures and ethnic groups. However the region covered reflects disproportionately more older people than the rest of the country and offers a unique mixture of care staff in different roles and positions and levels of leadership. Thus by drawing on combinations of front-line staff managers and informal carers it has been possible to explore nutritional care from multilevel perspectives of ‘what works’. As such the sampling approach was purposive for those organisations known for their ‘good practice’. We recognise that the present research did not seek the perspectives of people living with dementia in care homes and would require further investigation.

## Conclusions

In conclusion, the present study has identified key themes that have informed the development of a conceptual model to guide improvements in nutritional care by those responsible for the delivery of food and nutrition in dementia in nursing homes. Through this evidence-informed model we have since implemented new education and training tools (workbook and film) for caregivers to improve their knowledge, understanding and delivery of nutrition in dementia (www.bournemouth.ac.uk/nutrition-dementia). Further research is needed to evaluate the effectiveness of such evidence-based interventions and their impact on practice to directly support eating and drinking in people with dementia.

## Abbreviations

CS: Care staff
DN: Director of nursing
DT: Dietitian
FC: Family carer
HM: Hospitality manager
MUST: Malnutrition Universal Screening Tool
SLT: Speech and language therapist
